# Supplementary material for: Supervised breast cancer prediction using integrated dimensionality reduction convolutional neural network
Source: PLoS One. 2023 May 5;18(5):e0282350. doi: 10.1371/journal.pone.0282350 (PMC10162536; doi:10.1371/journal.pone.0282350)
Supplement: Attachment — Submitted filename: reponse letter.docx [file pone.0282350.s001.docx]

Dear Vijayalakshmi G V Mahesh and reviewers,

We would like to express our sincere gratitude to you for your efforts in handling our manuscript. We appreciate you give us the opportunity to revise our paper. We thank the editorial board’s and the two reviewer’s points and suggestions to improve our work. Those comments are all valuable and very helpful for revising and improving our paper.

We have studied comments carefully and have made correction which we hope meet with approval. Based on your comment and request, we attached revised manuscript with the correction sections red marked. We also replied the reviewers’ questions point-by-point as following below. The corrections in the paper and the responds to the reviewer’s comments are as following.

Should you have any questions, please contact us without hesitate.

**Reviewer #1:**

1. **There is limitation on statistical analysis of evaluation metrics from previous works**

**Response:** Thank you for your careful and constructive review and constructive comments. Our response is as follows:

The superiority of the proposed method is comprehensively evaluated by adding the original indexes such as Accuracy, Sensitivity, Specificity, PPV, and NPV. We have demonstrated that their algorithm is robust compared to existing algorithms. We have reworked the evaluation metric parameters in Table 4 by referring to other works found using the methods of others doing articles on the same breast tumour classification task compared to the method proposed in this paper. It is recorded that these results outperform the findings of other researchers in terms of evaluation metrics.

The confusion matrix is a table that shows the performance classifier. Generally, in machine learning, the confusion matrix is referred to as the error matrix. The image area may be positive or negative, depending on the data type. In addition, the decision on the detected result may be correct (true) or incorrect (false). Therefore, the decision will be one of four possible categories: true positive (TP), true negative (TN), false positive (FP) and false negative (FN). The correct decision is the diagonal of the confusion matrix.

$accuracy= \frac{TP+TN}{TP+FP+TN+FN}$ (3)

ROC analysis is a well-known method of evaluating detection tasks. Because of its common use in medical decision-making, ROC analysis is used in medical imaging.

The ROC curve is a graph of the operating point and can be regarded as a graph of the true false positive rate (FPR) and the true false positive rate (TPR). TPR and FPR are also called sensitivity and specificity, respectively, and are defined in formulas (4) and (5).

$$sensitivity= \frac{TP}{TP+FN} (4)$$

$$specificity= \frac{TN}{FP+TN} (5)$$

AUC is used in medical diagnostic systems for evaluating the model based on the average value of each point on the ROC curve. For classifier performance, the AUC score should always be between "0" and "1". A model with a higher AUC value has better classifier performance.

Precision refers to the number of positive observations correctly predicted relative to the total positive observations predicted. High precision is related to low FPR. Precision is calculated as shown in formula (6).

$precision= \frac{TP}{FP+TP}$	(6)

The F-value is a weighted average of the accuracy and recall. Therefore, this score considers both the false positive rate and the false positive rate. The F score is defined as shown in formula (7).

$$F-value = \frac{2*Recall*Precosion}{Recall+Precision} (7)$$

Positive Predictive Value (PPV) refers to the percentage of subjects who are judged to be positive (have cancer) by the early screening product. It is a measure of the ability of the early screening product to avoid "misdiagnosis" PPV = true positive population/positive test population. The ideal early screening product has a PPV of 100%, with no false positives.

$$PPV=\frac{TP}{TP+FP}(8)$$

Negative Predictive Value (NPV) refers to the percentage of subjects who are judged to be negative (healthy) for early screening products and is a measure of the ability of early screening products to "miss" and exclude negatives.

$$NPV=\frac{TN}{TN+FN}(9)$$

The improved model by the proposed algorithm was compared with several other classical models, and the experimental results are shown in Table 4 and Figures 9 and 10,11. It can be seen that the proposed model has better AUC, sensitivity, specificity, and accuracy compared to other deep learning models and machine learning models, indicating the feasibility of the model in the work of classifying breast cancer pathology images. After training, record the true positives, false positives, true negatives, and false negatives of the test set in turn.

**Table 4.** Evaluation index parameters.

| Methods | Accuracy  (AVG) | Sensitivity  (AVG) | Specificity  (AVG) | Precision (AVG) | F-value   (AVG) | PPV  (AVG) | NPV  (AVG) |
| --- | --- | --- | --- | --- | --- | --- | --- |
| CNN[31] | 0.81 | 0.67 | 0.80 | 0.68 | 0.67 | 0.77 | 0.83 |
| Machine learning[28] | 0.75 | 0.65 | 0.73 | 0.65 | 0.64 | 0.76 | 0.77 |
| Resnet50[30] | 0.83 | 0.81 | 0.81 | 0.75 | 0.79 | 0.81 | 0.79 |
| AlexNet[29] | 0.80 | 0.80 | 0.75 | 0.78 | 0.82 | 0.81 | 0.81 |
| Proposed model | **0.85** | **0.81** | **0.83** | **0.82** | **0.84** | **0.83** | **0.82** |

**Figure 9.** The accuracy of training dataset

**Figure 10.** The loss of training dataset

**Figure 11.** The auc of test dataset

In addition, ablation experiments were carried out to verify the modules' effectiveness, described below.

In the experiment part, ablation experiments were conducted on the data set of Breath Histology Images datasets for IDRCNN, CDCGAN, and Gaussian NB trees, and the following models were constructed for ablation experiments: (1) Gaussian NB trees were used to classify breast tumour images; (2) Gaussian NB tree combined with CDCGAN for breast tumour image classification; (3) Gaussian NB tree combined with CDCGAN and IDRCNN to classify breast tumour images; In order to ensure the fairness of the comparison experiment, the equipment and parameter settings used in the three groups of experiments are the same. The experimental results are shown in Figure 16 and Table 6.

**Table 6.** ablation experiments

| **Gaussian NB tree** | **CDCGAN** | **IDRCNN** | **Accuracy↑**  **(AVG)** | **Sensitivity↑**  **(AVG)** | **Specificity↑**  **(AVG)** |
| --- | --- | --- | --- | --- | --- |
| √ |  |  | 0.78 | 0.69 | 0.71 |
| √ | √ |  | 0.82 | 0.76 | 0.79 |
| √ | √ | √ | **0.85** | **0.81** | **0.83** |

**Figure 16.** The Results of the ablation experiment

Added version in lines 386-396 (Results): We demonstrate that other algorithms in the literature are robust compared to our algorithm. In Table 4, we add the evaluation metrics Accuracy, Sensitivity, Specificity, PPV, NPV, Precision, F-value and Auc to the evaluation parameters to fully evaluate the superiority of the proposed method.

2. **The similarity check between the real and fake images are not presented**

**Response:** Thank you for your careful and constructive review and constructive comments. Our response is as follows:

In this study, PSNR (peak signal-to-noise ratio), SSIM (structural similarity) and loss function are used to evaluate the quality of the generated samples, and a larger PSNR value indicates a better quality of the generated samples. A larger SSIM value indicates a better visual effect of the generated samples. Table 5 shows the PSNR and SSIM evaluation values.

**Table 5**. The PSNR and SSIM evaluation values.

| Method | PSNR( dB) | SSIM |
| --- | --- | --- |
| GAN | 22.36 | 0.7632 |
| DCGAN | 24.44 | 0.8033 |
| CDCGAN | 27.35 | 0.8422 |

As can be seen from Table 5 and Figures 12, 13, 14, and 15, the GAN loss function converged steadily at 100 iterations. The generated breast tumour slice samples were ambiguous because the GAN generative adversarial network training did not thoroughly learn the breast tumour sample features, with a PSNR value of 22.36 dB and SSIM of 0.7632. DCGAN converged faster than the GAN model, indicating that using a deep convolutional neural network accelerated the model training speed and improved the model feature extraction capability. The faster convergence speed of DCGAN compared to the GAN model indicated that the use of a deep convolutional neural network accelerated the training speed of the model and improved the feature extraction ability of the model, which further improved the ability of DCGAN to generate samples with PSNR value of 24.44 dB and SSIM value of 0.8033. The generated breast tumour samples had more apparent target edges and better visual effects with higher quality. The CDCGAN adds a conditional mechanism to the DCGAN to further focus on the practical breast tumour sample features, resulting in more precise details of the generated breast tumour texture with a PSNR of 27.35 dB and SSIM of 0.8422. This further illustrates the effectiveness of the CDCGAN conditional deep convolutional neural network proposed in this study.

**Figure 12.** The PSNR of Training dataset

**Figure 13.** The SSIM of Training dataset

**Figure 14.** The loss of Training dataset

**Figure 15.** The result of generate images in different models

Added version in lines 397-430 (Results): We have proposed a similarity check between true and false images and have used experiments to verify the superiority of our proposed method.

3. **Since the paper has work related on traditional machine learning, the features,the training samples and test samples considered for traditional machine learning are not presented.**

**Response:** Thank you for your careful and constructive review and constructive comments. Our response is as follows:

1) Pre-processing: the purpose of pre-processing is mainly to eliminate disturbing factors and highlight feature information.

The primary approach is as follows: the machine learning samples are normalized by the pre-processing image algorithm to eliminate the influence of other transformation functions on the image transformation, using the Z-Score algorithm, whose formula is shown below.

$$\begin{matrix} \text{image }_{\text{normalization} }=\frac{\text{image}-\mu}{\sigma} \\ \\ \text{ } \end{matrix}$$

The images are then subjected to data augmentation, and the amount of data is increased by rotation, translation and scaling, which can effectively prevent the overfitting problem of the machine learning model. In this paper, the rotation and translation and scaling operations are performed by setting the random rotation angle to [0-10 degrees], the random horizontal offset of the image to [0-0.1], the random vertical offset of the image to [0-0.1], and the random scaling to [0.9,1.1]. After the above geometric transformation makes, the breast tumour image data is augmented, increasing the training set samples and avoiding the model's overfitting problem due to insufficient training samples.	The data augmentation images are shown below. The first row shows the images before regularization, and the second row shows the images after regularization. The images of the data augmentation are shown below.

**Figure 5**. Data augmentation

2) Feature extraction: feature extraction of images using special feature extraction algorithms.

In this paper, we use a Histogram of Oriented Gradient (HOG) to extract features from breast tumour images, a statistical-based feature extraction algorithm: the feature vector of the image is obtained by counting the pixels with different gradient directions. The first row shows the regularized image, and the second row shows the image after extracting features by hog, as shown below:

**Figure 6**. Histogram of Oriented Gradient Feature Extraction

3) Feature processing. The primary purpose is to exclude features with little information and reduce the computation. The feature processing method used in this paper is the principal-form analysis method, which is to reduce the dimensionality of the breast image features and obtain the reduced features, and then put them into the machine learning model can reduce the computation.
4) Cross-validation. The training samples are split between implementing 80% of the training set and 10% of the validation set, 10% of the test set is split, and the obtained features are used to train the machine learning classification model using 10-fold cross-validation and take the mean value to improve the generalization ability of the model.

**Figure 8**. Ten-fold cross-validation schematic

Added version in line 301-385 (Results): This paper details the features, training samples and test samples considered in traditional machine learning.

4. **The papers has not mentioned on any other training options such as learning rate**

**Response:** Thank you for your careful and constructive review and constructive comments. Our response is as follows:

The experimental platform in this paper is a server with NVIDIA GeForce RTX 1080ti GPU and 16 GB memory, Windows system, Python 3.7, and the model training is done in pycharm 64-bit operating system. The experimental model is selected from tensorflow as the learning framework, which has advantages over other frameworks in terms of flexibility, ease of use, and speed3. The images are scaled to a uniform 50×50 and normalized. The processed images are divided into a training set, validation set and test set in the ratio of 8:1:1. In the experiment, all hyperparameters, including batchsize and learning rate, were used to determine the best performance of the classification model on the validation data set after several experiments with 100 training iterations, i.e., epochs of 100, and the best performance of the model on the validation data set was determined as follows: batchsize of 32, a learning rate of 0.001 is the minimum loss value. as shown in the following figure 7. The learning rate update strategy used is a natural exponential decay method.

**Figure 7**. Losses at different learning rates

Added version in line 341-372(Results):

The network model is trained to achieve the optimal classification of pathological images. The model is initialized by adjusting the input format, batch size, and network learning rate. The IDRCNN model from deep learning is applied to the detection of pathological images of breast cancer. The cross-entropy loss function is selected, and the IDRCNN network structure is modified with some network training techniques to train a suitable model for pathological images.

1) Selection of batch_size

The selection strategy for batch size requires consideration of computer memory and GPU memory resources, the dataset's sample size, and the input images' size. For large batches of training scenarios, it is essential to ensure that more appropriate batch size is used to avoid overflowing memory. The final batch size was determined to be 100 through empirical comparison.

2）Selection of learning_rate

The learning_rate is an essential parameter in deep learning that determines whether and when the objective function converges to a local minimum. A suitable learning rate allows the target function to converge to a local minimum in a suitable amount of time. The results are shown in Figure 7. The results are shown in Figure 7. As seen from Figure 7, the loss decreases as the learning_rate decreases, i.e. the error decreases. A significant learning rate will result in skipping the optimal point and obtaining a locally optimal point, resulting in a low accuracy rate. In contrast, a small learning rate will result in a relatively low training speed but closer to the optimal point.

In order to achieve the optimal classification of pathological images, the network model was trained. The images were scaled to a uniform 50×50 and normalized into a training, validation, and test set in a ratio of 8:1:1. All experiment hyperparameters, including batch size and learning rate, were used. After several experiments with 100 training batches per training iteration, i.e. epochs of 100, it was determined that the best performance of the classification model on the validation data set was the combination of parameters with a batch size of 32 and a learning rate of 0.001, as shown in Figure 7. The learning rate update strategy used was the natural exponential decay method.

**Reviewer #2:**

1. **Authors need to demonstrate the robustness of their algorithm in comparison with existing algorithms. This can be done in the results section. It may be noted that, in Table 3 Evaluation Index Parameters, the results of proposed algorithm are mentioned. How these results are superior to findings of other researchers. Authors need to refer the similar works in terms of their results.**

**Response:** Thank you for your careful and constructive review and your constructive comments. We have demonstrated that their algorithm is robust compared to existing algorithms. We have reworked the metric evaluation parameters in Table 4 by referring to other works found using the methods of others doing articles on the same breast tumour classification task compared to the method proposed in this paper. It is recorded that these results outperform the findings of other researchers in terms of evaluation metrics.

**Table 4.** Evaluation index parameters.

| Methods | Accuracy  (AVG) | Sensitivity  (AVG) | Specificity  (AVG) | Precision (AVG) | G-value   (AVG) | PPV  (AVG) | NPV  (AVG) |
| --- | --- | --- | --- | --- | --- | --- | --- |
| CNN[31] | 0.81 | 0.67 | 0.80 | 0.68 | 0.67 | 0.77 | 0.83 |
| Machine learning[28] | 0.75 | 0.65 | 0.73 | 0.65 | 0.64 | 0.76 | 0.77 |
| Resnet50[30] | 0.83 | 0.81 | 0.81 | 0.75 | 0.79 | 0.81 | 0.79 |
| AlexNet[29] | 0.80 | 0.80 | 0.75 | 0.78 | 0.82 | 0.81 | 0.81 |
| Proposed model | **0.85** | **0.81** | **0.83** | **0.82** | **0.84** | **0.83** | **0.82** |

The improved model by the proposed algorithm was compared with several other classical models, and the experimental results are shown in Table 4 and Figures 9 and 10,11. It can be seen that the proposed model has better AUC, sensitivity, specificity, and accuracy compared to other deep learning models and machine learning models, indicating the feasibility of the model in the work of classifying breast cancer pathology images. After training, record the true positives, false positives, true negatives, and false negatives of the test set in turn.

**Figure 9.** The accuracy of training dataset

**Figure 10.** The loss of training dataset

**Figure 11.** The auc of test dataset

Added version in lines 386-396 (Results): We demonstrate that other algorithms in the literature are robust compared to our algorithm. In Table 4, we add the evaluation metrics Accuracy, Sensitivity, Specificity, PPV, NPV, Precision, F-value and Auc to the evaluation parameters to fully evaluate the superiority of the proposed method.

**2. In the data preprocessing section, authors mentioned general geometric transformations. It is required to explain the specific details of transformations used.**

**Response:** Thank you for your careful and constructive review and constructive comments. Geometric transformation refers to the translation, rotation, and mirroring. of breast tumour images. Usually, in the traditional cv field, many disturbances can cause problems with the angle of the image when humans take it. The image needs to be corrected, and this correction process is based on geometric transformations. In addition, in the hot field of deep learning, there are cases where the number of samples is small, and the training model needs to be better, so it is necessary to obtain more training samples by oneself. The principles of the different geometric transformations are similar, but the transformation matrices are somewhat different.

In this paper, the rotation and translation, and scaling operations are performed by setting the random rotation angle to [0-10 degrees], the random horizontal offset of the image to [0-0.1], the random vertical offset of the image to [0-0.1], and the random scaling of the image to [0.9,1.1]. After the above geometric transformation, the breast tumour image data was augmented, increasing the sample of the training set and avoiding the overfitting problem of the model due to insufficient training samples. The data augmentation is shown in Figure 5.

**Figure 5**. Data augmentation

Added version in lines 324-333 (Results): We have explained the specific details of the transformations used. Augmenting the image with data by rotating, panning, and scaling can effectively prevent the overfitting problem of machine learning models. In this paper, the rotation, translation and scaling operations are performed by setting the random rotation angle to [0-10 degrees], the random horizontal offset of the image to [0-0.1], the random vertical offset of the image to [0-0.1] and the random scaling of the image to [0.9,1.1]. After the above geometric transformation, the breast tumour image data is augmented, increasing the sample of the training set and avoiding the overfitting problem of the model due to insufficient training samples. The data-augmented images are shown in Figure 5.

**3. For validation of the results, does authors used a totally new dataset other than training and testing data? or any cross validation techniques are used? provide specific details on validation methods adopted.**

**Response:** Thank you for your careful and constructive review and constructive comments. Cross Validation is a standard method used in machine learning to build models and validate model parameters. As the name suggests, the data is used repeatedly, and the resulting sample data is sliced, diced, and combined into different training and testing sets. The training set is used to train the model, and the test set is used to evaluate the model.

In this paper, a ten-fold cross-validation technique is used to split the training samples to implement 80% of the training set and 10% of the validation set, 10% of the test set is split, and the obtained features are used to train the machine learning classification model using ten-fold cross-validation and take the mean value to improve the generalisation ability of the model.

The experimental results were validated using ten-fold cross-validation. The 10-fold cross-validation divides the dataset into 10 equal-sized parts, each time taking one part as the test set and the rest as the training set. The steps of the 10-fold cross-validation are 1) divide the dataset into 10 equal parts, each containing the same number of images; 2) select one part at a time as the test set and the rest of the dataset as the model input to train the model; 3) after training, record the true positives, false positives, true negatives and false negatives of the test set in turn; 4) take the average of the 10 records to obtain the average sensitivity and average specificity of the final test results. sensitivity and an average specificity of the final test results. The principle is shown in Figure 8.

In this paper, a ten-fold cross-validation technique is used to split the training samples to implement 80% of the training set and 10% of the validation set, and 10% of the test set is split. The obtained features are used to train the machine learning classification model using ten-fold cross-validation and take the mean value to improve the model's generalisation ability.

The experimental results were validated using ten-fold cross-validation. The 10-fold cross-validation divides the dataset into ten equal-sized parts, each taking one part as the test set and the rest as the training set. The steps of the 10-fold cross-validation are

1) divide the dataset into ten equal parts, each containing the same number of images;
2) select one part at a time as the test set and the rest of the dataset as the model input to train the model;
3) after training, record the true positives, false positives, true negatives and false negatives of the test set in turn;
4) take the average of the ten records to obtain the average sensitivity and average specificity of the final test results.

The principle is shown in Figure 8.

**Figure 8**. Ten-fold cross-validation schematic

Added version in lines 375-385 (Results): A ten-fold cross-validation method was used to validate the experimental results. The tenfold cross-validation divides the data set into ten equal-sized parts.

Each time, a part of the data set is taken as the test set and the rest as the training set, and the average of the ten results is taken as the model's metric estimation. The principle is shown in Figure 8, and the steps of ten-fold cross-validation are

1) Divide the dataset equally into ten parts, each containing the same number of images.

2) Select one part at a time as the test set and the rest of the dataset as model input to train the model.

3) After training, record the true positives, false positives, true negatives and false negatives of the test set in turn.

4) After training, record the true positives, false positives, true negatives, and false negatives of the test set in turn.

Once again, thank you very much for your constructive comments and suggestions which would help us both in English and in depth to improve the quality of the paper.

Yours sincerely,

Huanqing Xu,Xian Shao,Shiji Hui, Li Jin

2022/12/21
